# Efficacy of Zinc Supplementation in the Management of Primary Dysmenorrhea: A Systematic Review and Meta-Analysis

**DOI:** 10.3390/nu16234116

**Published:** 2024-11-28

**Authors:** Ting-Jui Hsu, Rong-Hong Hsieh, Chin-Huan Huang, Chih-Shou Chen, Wei-Yu Lin, Yun-Ching Huang, Jian-Hui Lin, Kuo-Tsai Huang, Yu-Liang Liu, Hui-Ming Tsai, Dong-Ru Ho

**Affiliations:** 1 Division of Urology, Department of Surgery, Chang Gung Memorial Hospital, Chiayi 61363, Taiwan; tony110681@cgmh.org.tw (T.-J.H.); cgmh5093@gmail.com (C.-S.C.); checotrade@gmail.com (W.-Y.L.); dr5326@cgmh.org.tw (Y.-C.H.); b9005026@gmail.com (J.-H.L.); ronsolglobalinc@gmail.com (K.-T.H.); armsliu@cgmh.org.tw (Y.-L.L.); 2 School of Nutrition and Health Sciences, College of Nutrition, Taipei Medical University, Taipei 11031, Taiwan; hsiehrh@tmu.edu.tw; 3 Research Center of Nutritional Medicine, College of Nutrition, Taipei Medical University, Taipei 11031, Taiwan; 4 Program in Drug Discovery and Development Industry, College of Pharmacy, Taipei Medical University, Taipei 11031, Taiwan; 5 Department of Nutrition Therapy, Dalin Tzu Chi Hospital, Buddhist Tzu Chi Medical Foundation, Chiayi 622401, Taiwan; dalinrd@tzuchi.com.tw; 6 School of Medicine, National Cheng Kung University, Tainan City 701401, Taiwan; 7 Graduate Institute of Clinical Medical Sciences, College of Medicine, Chang Gung University, Taoyuan 33302, Taiwan; 8 Department of Early Childhood Care and Education, Shu-Zen Junior College of Medicine and Management, Kaohsiung City 82144, Taiwan; 9 School of Medicine, National Tsing Hua University, Hsinchu 300044, Taiwan

**Keywords:** zinc supplementation, primary dysmenorrhea, menstrual pain, pain relief, anti-inflammatory, women’s health, randomized controlled trials, meta-analysis, nutritional supplements

## Abstract

Background/Objectives: Primary dysmenorrhea (PD) is a common condition affecting up to 90% of menstruating women, which often results in significant pain without an underlying pathology. Zinc, recognized for its anti-inflammatory and antioxidant effects through inhibiting prostaglandin production and superoxide dismutase 1 (SOD1) upregulation, alleviates menstrual pain by preventing uterine spasms and enhancing microcirculation in the endometrium, suggesting its potential as an alternative treatment for primary dysmenorrhea. The goal of this systematic review and meta-analysis was to assess the efficacy and safety of zinc supplementation in reducing pain severity among women with PD and to explore the influence of dosage and treatment duration. Methods: Following the PRISMA 2020 guidelines, we conducted an extensive search across databases such as PubMed, Embase, Cochrane Library, Web of Science, and Google Scholar, up to May 2024. Randomized controlled trials assessing the effects of zinc supplementation on pain severity in women with PD were included. Pain severity was evaluated with established tools, such as the Visual Analog Scale (VAS). Risk of bias was assessed using the Cochrane Risk of Bias 2 (RoB2) tool. Two reviewers independently performed the data extraction, and a random-effects model was used for meta-analysis. Meta-regressions were conducted to examine the influence of zinc dosage and treatment duration on pain reduction. Adverse events were also analyzed. Results: Six RCTs involving 739 participants met the inclusion criteria. Zinc supplementation significantly reduced pain severity compared to placebo (Hedges’s g = −1.541; 95% CI: −2.268 to −0.814; *p* < 0.001), representing a clinically meaningful reduction in pain. Meta-regression indicated that longer treatment durations (≥8 weeks) were associated with greater pain reduction (*p* = 0.003). While higher zinc doses provided additional pain relief, the incremental benefit per additional milligram was modest (regression coefficient = −0.02 per mg; *p* = 0.005). Adverse event rates did not differ significantly between the zinc and placebo groups (odds ratio = 2.54; 95% CI: 0.78 to 8.26; *p* = 0.122), suggesting good tolerability. Conclusions: Zinc supplementation is an effective and well-tolerated option for reducing pain severity in women with primary dysmenorrhea. Doses as low as 7 mg/day of elemental zinc are sufficient to achieve significant pain relief, with longer durations (≥8 weeks) enhancing efficacy. The favorable safety profile and ease of use support the consideration of zinc supplementation as a practical approach to managing primary dysmenorrhea.

## 1. Introduction

Primary dysmenorrhea (PD) is characterized by cramp-like pain in the lower abdomen that occurs just before or at the onset of menstruation, without any identifiable pelvic pathology. PD affects 45% to 95% of reproductive-aged women worldwide, with 2% to 29% reporting severe pain that can significantly impair daily activities [1]. Besides lower abdominal pain, PD is often associated with symptoms, including headache, back and thigh pain, fatigue, nausea, vomiting, and diarrhea [2,3]. The exact etiology of PD remains not entirely understood, but it is generally attributed to the excessive production of prostaglandin F2α (PGF2α) and leukotrienes in the endometrium during menstruation, which lead to intense or irregular uterine muscle contractions and vasoconstriction, resulting in uterine ischemia and pain [4,5,6].

Current management strategies for PD primarily involve the administration of non-steroidal anti-inflammatory drugs (NSAIDs) [7,8]. However, long-term use can lead to side effects, such as gastrointestinal disturbances, nausea, vomiting, and dizziness [3].

Given the substantial medical, social, and economic burden of PD, there is a pressing need to identify effective treatments with fewer side effects and better tolerability than current options.

Zinc, an essential trace element involved in numerous enzymatic reactions, has attracted attention for its potential role in reproductive health [9,10]. Zinc has been shown to modulate cyclooxygenase activity and inhibit prostaglandin synthesis, to enhance microcirculation and prevent ischemia in uterine tissues, and to exhibit antioxidant properties by increasing superoxide dismutase levels and downregulating inflammatory cytokines [10,11].

Recent studies have suggested that zinc supplementation can significantly reduce the severity and duration of dysmenorrhea symptoms compared to placebo [12,13]. Additionally, Nasiadek et al. (2020) [9] emphasized the importance of zinc in disorders of the female reproductive system, highlighting its importance in modulating inflammatory responses and hormonal regulation, which are critical factors in the pathogenesis of PD.

Considering the increasing evidence supporting the beneficial effects of zinc on PD, a systematic review and meta-analysis are warranted to evaluate the efficacy and safety of zinc supplementation for managing primary dysmenorrhea. This study aims to assess the impact of zinc supplementation on pain severity and associated symptoms, analyze the influence of dosage and treatment duration, and provide reliable evidence to inform clinical practice.

## 2. Materials and Methods

### 2.1. General Guidelines

This systematic review and meta-analysis adhered to the Preferred Reporting Items for Systematic Reviews and Meta-Analyses (PRISMA) 2020 guidelines Supplementary Table S1 [14]. The study protocol was registered in PROSPERO with the registration number CRD42024579213 [15]. No ethical approval or informed consent was required since the study utilized previously published data.

### 2.2. Search Strategy and Selection of Eligible Studies

Two independent authors (T.-J.H. and H.-M.T.) performed extensive database searches in the PubMed, Cochrane Library, Embase, Web of Science, and Google Scholar databases using the following keywords: (“Zinc Supplementation” OR “Zinc therapy” OR “Zinc sulfate” OR “Oral zinc”) AND (“Primary Dysmenorrhea” OR “Menstrual pain” OR “Menstrual cramps” OR “Period pain” OR “Dysmenorrhoea”) AND (“Randomized Controlled Trial” OR “Clinical trial” OR “RCT” OR “Randomised trial”). The search covered all publications up to 24 August 2024. Additionally, we performed a hand search of the reference lists of relevant articles to find any other eligible studies. Studies published in English were included. The detailed search strategy is provided in the Supplementary Materials Table S2.

### 2.3. Inclusion and Exclusion Criteria

The meta-analysis was structured using the PICO (Population, Intervention, Comparison, Outcome) framework: Population (P): women diagnosed with primary dysmenorrhea; Intervention (I): zinc supplementation alone or combined with other treatments (e.g., NSAIDs), in any form and dosage; Comparison (C): placebo, or other treatments (e.g., NSAIDs) plus placebo; and Outcome (O): outcomes related to pain severity.

The criteria for inclusion were as follows: (1) randomized controlled trials (RCTs), (2) studies evaluating the efficacy of zinc supplementation in primary dysmenorrhea, and (3) studies reporting outcomes related to pain severity.

The criteria for exclusion included the following: (1) studies involving secondary dysmenorrhea; (2) observational studies, reviews, and case reports; and (3) studies with incomplete data or non-comparable outcomes.

The detailed inclusion and exclusion criteria, along with their explanations, are provided in the Supplementary Materials Table S3.

### 2.4. Methodological Quality Appraisal

We evaluated the quality of the methodologies in the selected studies using the Cochrane Risk of Bias tool for randomized trials (version 2, RoB 2) [16]. This tool evaluates six domains: the randomization process, deviations from intended interventions, missing outcome data, measurement of the outcome, selection of the reported result, and overall risk of bias. Two reviewers (T.-J.H. and H.-M.T.) independently assessed each study, and any disagreements were addressed through discussion or by consulting a third reviewer (R.-H.H).

### 2.5. Primary Outcome

The primary outcome measured was the change in pain severity associated with primary dysmenorrhea, assessed using validated pain scales like the Visual Analog Scale (VAS) [17] or the Numeric Rating Scale (NRS) [18]. If multiple pain assessments were reported, the most commonly used scale across studies was selected for analysis.

### 2.6. Secondary Outcome

The secondary outcome examined in this analysis was the incidence of adverse events related to the treatment. Odds ratios were used to quantify these outcomes.

### 2.7. Data Extraction and Management

Two independent reviewers (T.-J.H. and H.-M.T.) gathered data through a standardized form. The extracted details encompassed study characteristics (authors, year of publication, and country), participant demographics, intervention details (zinc dosage, form, and duration), comparison interventions, outcome measures, and results. Any inconsistencies were settled by reaching an agreement or by involving a third reviewer. When necessary, we contacted study authors for additional information or clarification.

### 2.8. Statistical Analyses

The statistical evaluations were conducted using Comprehensive Meta-Analysis software (version 3.7; Biostat, Englewood, NJ, USA). For continuous outcomes, we calculated Hedges’s g with 95% confidence intervals (CIs). Hedges’s g values of 0.2, 0.5, and 0.8 were considered to represent small, moderate, and large effect sizes, respectively [19]. For dichotomous outcomes, odds ratios (ORs) with 95% CIs were determined.

The variability among studies was evaluated using the *I*^2^ statistic and Cochran’s Q test. *I*^2^ statistics of 25%, 50%, and 75% were interpreted as indicating low, moderate, and high heterogeneity, respectively [20]. Due to the anticipated differences among studies, a random-effects model was applied [21].

Subgroup analyses were carried out based on treatment duration. Meta-regression analyses were performed to investigate the association between treatment effects and factors such as daily zinc dosage and the length of supplementation.

Sensitivity analysis was conducted using the one-study removal method to determine whether excluding any single trial would result in a statistically significant change in the overall effect size. Publication bias was assessed through funnel plots and Egger’s regression test. Egger’s regression test was applicable when the meta-analysis included ten or more studies [22].

## 3. Results

### 3.1. Study Identification and Selection

**Figure 1:**
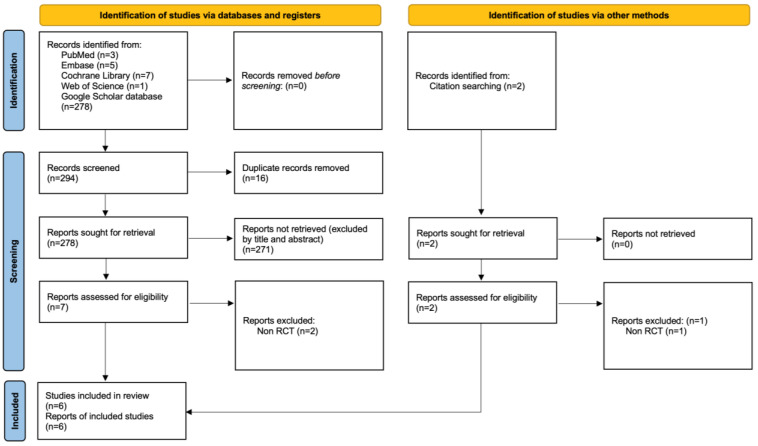
PRISMA 2020 diagram summarizing the study selection process for this meta-analysis.

The PRISMA diagram detailing the procedures for literature searching and study selection is displayed in Figure 1. After eliminating duplicate entries and reviewing titles and abstracts, we identified six randomized controlled trials (RCTs) that fulfilled the inclusion criteria for this meta-analysis [12,13,23,24,25,26]. The articles that were excluded in the final stage, along with the reasons for their exclusion, are listed in the Supplementary Materials Table S4. The detailed data extracted from the included trials are summarized in the Supplementary Materials Table S5. The features of the studies included in this analysis are summarized in Table 1. The intervention details, dosage of zinc supplements, tools for pain assessment, and study withdrawals are summarized in Table 2.

The six included RCTs encompassed a total of 739 participants aged between 13 and 27 years, all of whom were women diagnosed with primary dysmenorrhea. The studies were conducted in different countries, including Iran, Nigeria, and Pakistan, and had treatment durations ranging from one to six months. However, these trials did not report participants’ dietary zinc intake, zinc status, or overall nutritional status, such as BMI or biochemical markers. Therefore, we are unable to determine the nutritional status of the participants or whether they had zinc deficiency.

### 3.2. Methodological Quality of the Included Studies

**Figure 2:**
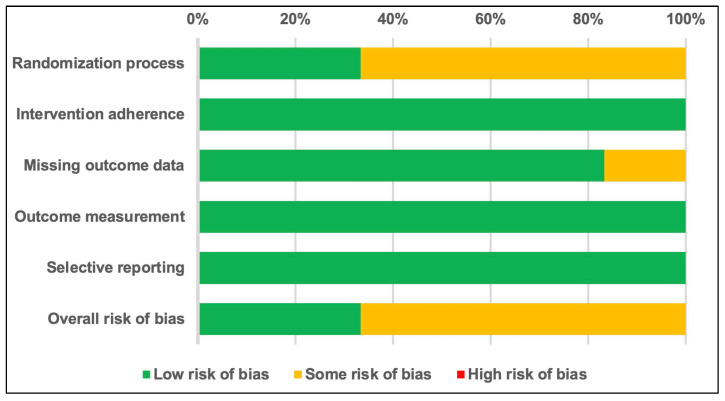
Quality assessment summary for studies included in the meta-analysis, evaluated using the Cochrane Risk of Bias 2 tool.

The methodological quality of the included studies was assessed using the Cochrane Risk of Bias tool for randomized trials (RoB 2) [16]. The overall risk of bias was mixed: 33% of the studies were rated as having a low risk of bias, and 67% had some concerns Figure 2. Common issues included insufficient details on randomization and allocation concealment, as well as insufficient handling of missing outcome data in some studies. Detailed assessments are provided in Table 3.

### 3.3. Primary Outcome: Effects of Zinc Supplementation on Pain Severity

**Figure 3:**
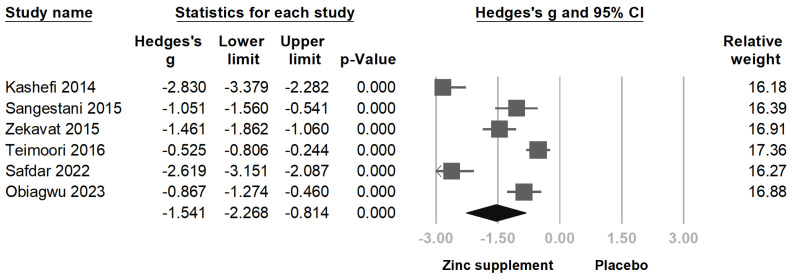
Forest plot showing the overall effect of zinc supplementation on pain severity using the longest follow-up data [12,13,23,24,25,26].

Using the longest follow-up data from each study, we performed a meta-analysis to evaluate the effectiveness of zinc supplementation in reducing pain severity among women with primary dysmenorrhea. The pooled analysis of all six studies demonstrated a statistically significant reduction in pain severity with zinc supplementation compared to placebo (Hedges’s g = −1.541; 95% CI = −2.268 to −0.814; *p* < 0.001; *I*^2^ = 94.31%; Q = 87.918) Figure 3. The effect size indicates a large reduction in pain severity associated with zinc supplementation.

Given the high heterogeneity observed among the studies (*I*^2^ = 94.31%), we explored possible contributors to heterogeneity through subgroup analyses and meta-regressions.

**Figure 4:**
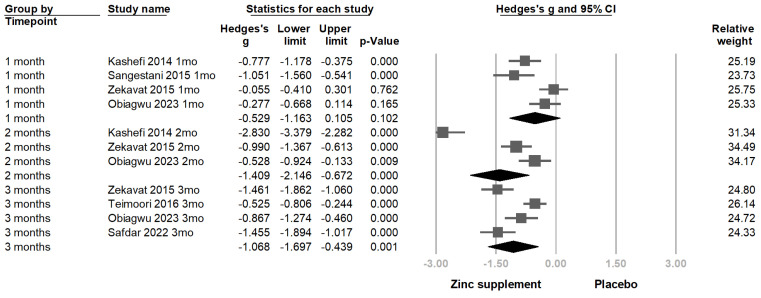
Forest plot for the subgroup analysis using the follow-up duration as the moderator, including one, two, and three months. The directions of association between zinc supplementation and pain scale assessment were consistent across all follow-up durations, with overlapping 95% confidence intervals (CIs), indicating similar trends in pain reduction over time despite varying levels of statistical significance and heterogeneity [12,13,23,24,25,26].

We first performed a subgroup analysis based on follow-up duration Figure 4.

At the 1-month follow-up, four studies [12,23,24,25] reported outcomes. The pooled effect size was Hedges’s g = −0.529 (95% CI = −1.163 to 0.105; *p* = 0.102; *I*^2^ = 77.6%). This indicates a moderate reduction in pain severity at 1 month with zinc supplementation; however, the result did not reach statistical significance at the 0.05 level.

At the 2-month follow-up, three studies [12,23,25] provided data. The pooled effect size was Hedges’s g = −1.409 (95% CI = −2.146 to −0.672; *p* < 0.001; *I*^2^ = 95.7%), suggesting a large reduction in pain severity at 2 months.

At the 3-month follow-up, four studies [12,13,25,26] reported outcomes. The pooled effect size was Hedges’s g = −1.068 (95% CI = −1.697 to −0.439; *p* = 0.001; *I*^2^ = 85.2%), demonstrating a sustained large reduction in pain severity at 3 months.

**Figure 5:**
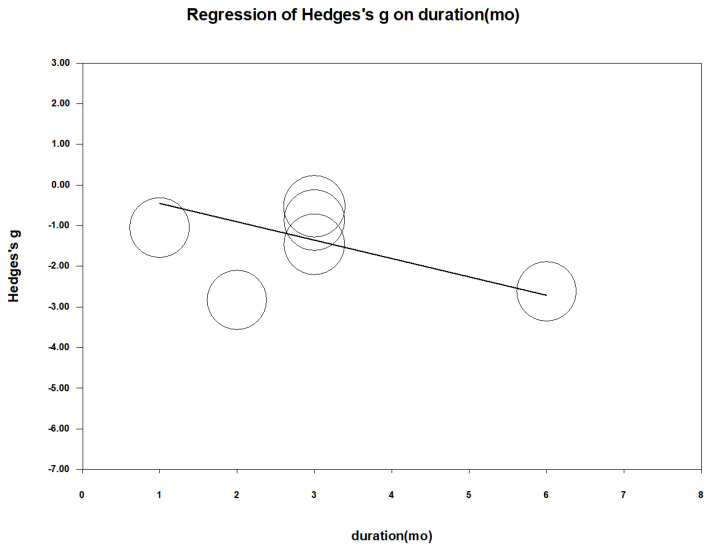
Meta-regression of Hedges’s g versus treatment duration (month). The coefficient was −0.452 with a *p* value < 0.001.

To investigate the relationship between treatment duration and effect size, a meta-regression was performed using follow-up time (in months) as a continuous moderator. The analysis revealed a significant association between longer treatment duration and greater reductions in pain severity (regression coefficient = −0.452 per month; *p* < 0.001) Figure 5. This suggests that the efficacy of zinc supplementation increases with longer treatment periods.

**Figure 6:**
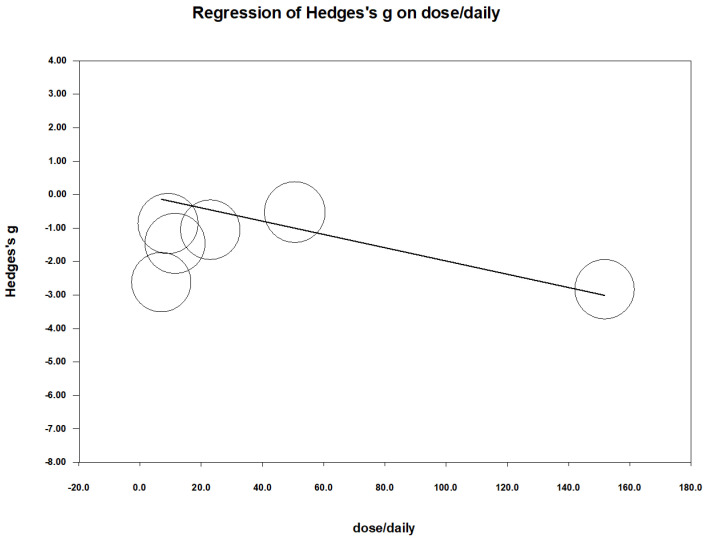
Meta-regression of Hedges’s g against daily dose (mg/day). Coefficient = −0.02; *p* = 0.005.

We also conducted a meta-regression to examine the impact of zinc dosage (milligrams per day) on pain reduction. To ensure accurate dosing comparisons, we converted all zinc supplement dosages to elemental zinc based on their specific compositions, referencing Saper et al. (2009) [27], which states that zinc gluconate contains 14.3% elemental zinc and zinc sulfate contains 23% elemental zinc. The analysis indicated a significant dose–response relationship, with higher daily doses of zinc associated with greater reductions in pain severity (regression coefficient = −0.02 per milligrams; *p* = 0.005) Figure 6. This finding implies that both the duration and dosage of zinc supplementation contribute to its effectiveness in alleviating menstrual pain.

**Figure 7:**
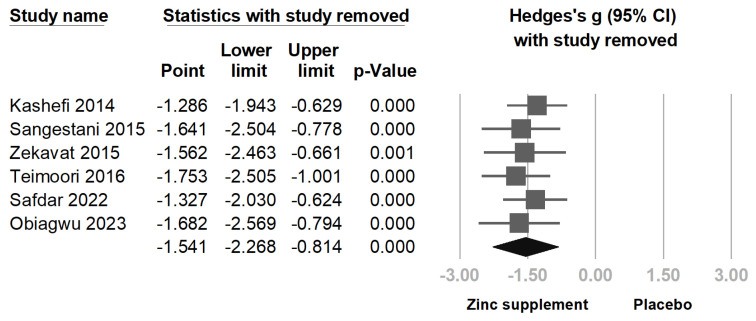
Sensitivity analysis using the one-study removal method. The primary outcome remained consistent after excluding each individual trial. Zinc supplementation consistently showed significant pain reduction [12,13,23,24,25,26].

To assess the robustness of our findings and further explore heterogeneity, sensitivity analysis was undertaken through the one-study removal method Figure 7. Excluding Kashefi et al. (2014) [23], which had the largest effect size and contributed substantially to heterogeneity, reduced the *I*^2^ value to 92.2% and yielded a pooled effect size of Hedges’s g = −1.286 (95% CI = −1.943 to −0.629; *p* < 0.001). This indicates that while heterogeneity decreased, the overall significant effect of zinc supplementation on pain reduction remained consistent.

**Figure 8:**
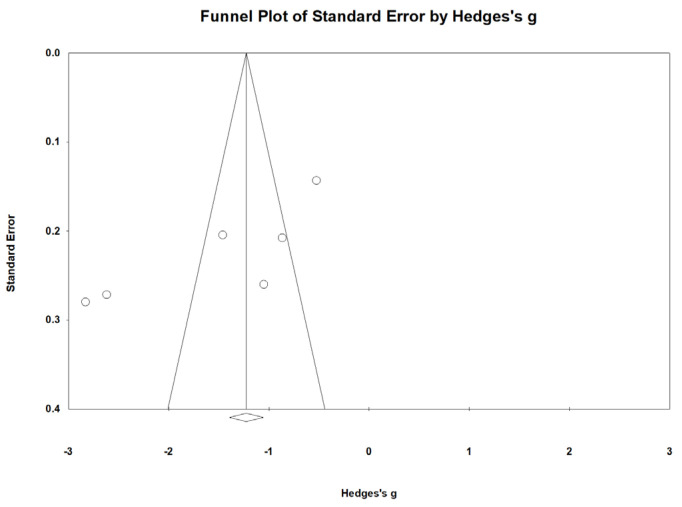
Funnel plot of included studies displaying asymmetrical distribution, suggesting potential publication bias.

To evaluate the potential for publication bias, a funnel plot of effect sizes versus standard errors was created Figure 8. Visual assessment revealed asymmetry, suggesting potential publication bias.

### 3.4. Secondary Outcome: Treatment-Associated Adverse Events

**Figure 9:**
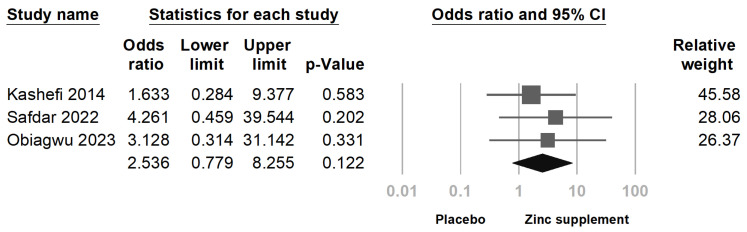
Forest plot of adverse event rates associated with treatment. No significant difference was observed between the two groups (*p* = 0.122) [12,13,23].

The incidence of treatment-associated adverse events was low across the included studies. Only 11 participants reported mild side effects, such as gastrointestinal discomfort and nausea [12,13,23]. The meta-analysis of adverse event rates showed no statistically significant difference between the zinc supplementation group and the placebo group (odds ratio [OR] = 2.536; 95% CI = 0.78 to 8.26; *p* = 0.122; *I*^2^ = 0%) Figure 9. This suggests that zinc supplementation is generally well-tolerated among women with primary dysmenorrhea.

## 4. Discussion

### 4.1. Summary of Findings

In this meta-analysis, zinc supplementation was shown to significantly reduce pain severity in women with primary dysmenorrhea, and this statistical significance was maintained in sensitivity analyses. We found that lower doses of zinc were sufficient to achieve therapeutic outcomes without increasing the risk of adverse events. Moreover, longer durations of zinc supplementation correlated with greater reductions in pain severity. To our understanding, this study is the first systematic review and meta-analysis to quantify the effect of zinc on primary dysmenorrhea.

### 4.2. Mechanisms of Zinc in Dysmenorrhea

The pathogenesis of primary dysmenorrhea is closely linked to elevated levels of prostaglandins and leukotrienes. Studies have demonstrated that women experiencing painful menstruation exhibit higher concentrations of PGF2-alpha and leukotrienes in their menstrual blood and uterine tissues [2,5,28]. These mediators induce strong uterine contractions, which temporarily reduce or halt blood flow to the uterus, leading to oxygen deprivation, muscle spasms, and resultant pain [29]. Furthermore, ischemia–reperfusion injury contributes to dysmenorrhea, as the restoration of blood flow generates reactive oxygen species (ROS) that cause tissue damage and exacerbate pain. Consequently, the presence of these free oxygen radicals likely intensifies the discomfort associated with dysmenorrhea [30].

Zinc supplementation may alleviate these symptoms through multiple mechanisms. Primarily, zinc reduces prostaglandin synthesis via its antioxidant and anti-inflammatory effects, thereby improving microcirculation in the endometrium [10,31,32,33]. Enhanced uterine circulation can mitigate ischemia and reperfusion injury, subsequently decreasing the release of ROS that cause tissue damage and pain [11,30]. In vivo studies have indicated that zinc decreases the activity of cyclooxygenase-2 (COX-2), a key enzyme involved in prostaglandin production [32,34]. Additionally, zinc may prevent uterine spasms and pain by modulating oxidative stress and inflammatory responses, potentially through the upregulation of superoxide dismutase 1 (SOD1) and the reduction of inflammatory cytokines [35,36].

The presence of copper–zinc superoxide dismutase in uterine tissue further supports zinc’s role in enhancing antioxidant defenses, which may alleviate cramping and pain [37]. Moreover, lower zinc levels have been observed in women with premenstrual syndrome during the luteal phase compared to controls, suggesting a potential zinc deficiency [38]. This deficiency may contribute to the increased severity of dysmenorrhea symptoms, highlighting the importance of adequate zinc supplementation.

### 4.3. Clinical Evidence Supporting Zinc’s Therapeutic Effects

Recent clinical studies have further supported the therapeutic effects of zinc on menstrual-related symptoms. Jafari et al. (2020) [39] performed a randomized, double-blind, placebo-controlled trial and revealed that zinc supplementation significantly reduced physical and psychological symptoms in young women with premenstrual syndrome (PMS) by decreasing biomarkers of inflammation and oxidative stress. Similarly, Ahmadi et al. (2023) [40] reported that zinc supplementation resulted in significant improvements in PMS symptoms among female university students, highlighting zinc’s role in modulating inflammatory responses. Additionally, Oboza et al. (2024) [41] explored the relationships between PMS and dietary factors, finding that zinc consumption correlated with a decrease in PMS symptoms, which suggests a potential link between zinc intake and menstrual pain relief. However, previous reviews did not quantify the analgesic effect of zinc specifically on menstrual pain. Our meta-analysis addresses this gap by providing a quantitative assessment of zinc’s impact on pain severity in dysmenorrhea, offering more definitive evidence of its effectiveness as an analgesic agent in this context.

### 4.4. Other Nutrients in Managing Dysmenorrhea

In addition to zinc, several other nutrients have shown promising results in managing dysmenorrhea and other inflammatory conditions. Studies indicate that vitamin D can reduce the severity of menstrual pain by decreasing the synthesis of prostaglandins, which are key mediators of pain and inflammation [42,43,44]. Furthermore, calcium has been shown to reduce muscle contractions, which can alleviate the intensity of menstrual cramps [42,43,45,46]. Recent findings suggest that curcumin, a polyphenol found in turmeric, demonstrates significant anti-inflammatory effects by suppressing the synthesis of prostaglandins [47], thereby helping to reduce menstrual pain [48,49,50]. In a randomized controlled trial by Talebpour et al. (2023) [51], curcumin supplementation significantly decreased high-sensitivity C-reactive protein (hsCRP), an inflammatory biomarker, without affecting iron metabolism in healthy women with PMS and dysmenorrhea. Based on the above evidence, curcumin may reduce the severity of dysmenorrhea and premenstrual syndrome (PMS) symptoms.

Zinc, vitamin D, and curcumin all reduce prostaglandin synthesis through anti-inflammatory processes, thereby decreasing pain severity. In contrast, calcium alleviates menstrual pain by directly reducing uterine muscle contractions, representing a different pathway to pain relief. Consistent with previous reviews, vitamin D supplementation typically requires approximately two months to manifest positive effects [42,52,53], aligning with our study’s findings of significant symptom relief after an eight-week intervention period.

However, there is insufficient research in the literature to directly compare the effects of these nutrients with zinc on primary dysmenorrhea or to comprehensively assess their combined effects and side-effect profiles. While the concurrent use of these nutrients may offer enhanced efficacy in alleviating pain and improving overall well-being, further research is necessary to confirm their synergistic benefits and to evaluate the potential for increased adverse effects, such as gastrointestinal disturbances.

It is possible that combining zinc and calcium could provide additional therapeutic benefits due to their distinct mechanisms of action. Nonetheless, clinical trials are required to establish their efficacy and safety when used together.

The combined use of these nutrients may be more effective than using them individually in alleviating pain and improving overall well-being. Future research should explore their synergistic effects to provide a comprehensive, non-pharmacological approach to managing dysmenorrhea and similar inflammatory conditions.

### 4.5. Addressing High Heterogeniety in the Meta-Analysis

While our findings on zinc supplementation offer insight into a potential non-pharmacological option for managing primary dysmenorrhea, the high level of heterogeneity (*I*^2^ = 94.31%) among included studies warrants careful consideration. This substantial variability likely stems from differences in study protocols, including variations in zinc formulations, dosages, and treatment durations, which may affect the overall effectiveness of zinc in pain reduction. For example, the study by Kashefi et al. (2014) [23] used a considerably higher dose of zinc sulfate, while the study by Safdar et al. (2022) [13] utilized a different form of supplement—zinc gluconate at a lower dose—to the other five studies, which used zinc sulfate and included extended follow-up periods of 3 to 6 months. These factors contribute to the observed heterogeneity, underscoring the need for a cautious interpretation of our pooled results and suggesting that standardized approaches in future studies would be beneficial.

Given these differences, we conducted further analyses to explore the specific effects of zinc dosage and treatment duration on its efficacy, aiming to clarify how these factors influence outcomes and to provide a more comprehensive understanding of zinc’s role in alleviating primary dysmenorrhea.

### 4.6. Impact of Zinc Dosage and Treatment Duration

In our subgroup analysis based on follow-up duration, we observed that at the 1-month follow-up, the pooled effect size was Hedges’s g = −0.529 (*p* = 0.102), which was not statistically significant. This suggests that zinc supplementation for one month may not provide significant pain relief in primary dysmenorrhea. However, at the 2-month and 3-month follow-ups, the pooled effect sizes were Hedges’s g = −1.409 and Hedges’s g = −1.068, respectively (both *p* < 0.05), indicating large and statistically significant reductions in pain severity with zinc supplementation over these longer durations.

Interestingly, the effect size at 2 months was greater than at 3 months. This unexpected finding may be attributed to the study by Kashefi et al. (2014) [23], which contributed the largest individual effect size to the analysis at the 2-month mark (Hedges’s g = −2.83). The substantial impact of this single study likely inflated the pooled effect size at 2 months, resulting in a greater effect estimate compared to the 3-month follow-up. This highlights the influence that individual studies with large effect sizes can have on meta-analytic results, especially when the number of included studies is limited [54]. Nevertheless, these findings underscore the importance of sustained zinc supplementation for achieving significant pain relief in primary dysmenorrhea, with noticeable benefits emerging after at least two months of treatment.

Building on this observation, our meta-regression demonstrated a positive relationship between treatment duration and pain reduction. Specifically, longer durations of zinc supplementation were associated with greater decreases in pain severity. In the reviewed studies, women were supplemented with zinc for periods ranging from 3 to 7 days [12,13,23,24,26], mostly spanning two to three menstrual cycles. While some reduction in pain was observed during the first cycle of zinc supplementation, the most pronounced effects were evident in the second and third cycles [12,23,25]. Importantly, the decrease in pain severity was consistent across various zinc dosages (7 to 150 mg/day) administered for 3–7 days either before or during the menstrual cycle. These findings suggest that the sustained and regular intake of zinc, rather than the specific dosage, plays a pivotal role in mitigating menstrual pain associated with primary dysmenorrhea.

### 4.7. Safety Considerations

Safety is a critical consideration in managing primary dysmenorrhea, especially for long-term treatment. One of the included studies, Kashefi et al. (2014) [23], utilized zinc sulfate at a dosage of 220 mg taken three times daily. According to Saper et al. (2009) [27], 220 mg of zinc sulfate contains approximately 50 mg of elemental zinc. Therefore, participants in this study received a total daily intake of about 150 mg of elemental zinc, significantly exceeding the tolerable upper intake level (UL) for zinc, which is set at 40 mg per day for adults by the United States National Academy of Medicine [55]. Exceeding the UL may lead to adverse effects, such as gastrointestinal discomfort, nausea, vomiting, and disruptions in copper metabolism, potentially resulting in copper deficiency [56,57].

Conversely, more recent research, such as the randomized controlled trial by Safdar et al. (2022) [13], has shown that lower doses of zinc supplementation can effectively improve primary dysmenorrhea symptoms. In their study, a daily dose of 50 mg of zinc gluconate (equivalent to approximately 7 mg of elemental zinc [27]), which is within the recommended dietary allowance (RDA) of 8 mg/day for adult women set by the National Academy of Medicine [55], was effective in reducing menstrual pain. Although higher doses are associated with greater pain reduction, our meta-regression analysis of dosage per day indicates that the incremental benefit per additional milligram is modest; for example, increasing the dose by 10 mg/day leads to only a 0.2 improvement in effect size, corresponding to a small reduction in pain levels. Therefore, optimal pain relief can be achieved with lower doses of zinc, minimizing the potential for dose-related side effects.

### 4.8. Limitations

This study presents several limitations. First, the number of included studies was relatively small—six RCTs with a total of 739 participants—which may affect the statistical power and limit the generalizability of the findings. Additionally, the existence of publication bias, as suggested by the asymmetry in the funnel plot, indicates that studies with non-significant results might be underrepresented.

Second, the included trials did not report detailed information on the participants’ dietary zinc intake; zinc status assessed through laboratory measures; or overall nutritional status, such as BMI or biochemical markers. This lack of individual zinc status and nutritional data limits our ability to assess the potential role of baseline zinc deficiency in modulating the efficacy of zinc supplementation on primary dysmenorrhea.

Moreover, most of the included studies were conducted in regions where zinc deficiency is more prevalent—four in Iran, one in Pakistan, and one in Nigeria. According to Brown et al. (2001) [58], countries in the Middle East, North Africa, and parts of Asia and Africa tend to have lower zinc intake levels and a higher risk of zinc deficiency. The study indicates that the food supplies in these regions provide only about 50 to 60% of the normative physiological zinc requirements, with 68 to 95% of the population potentially at risk of inadequate zinc intake due to the low availability of absorbable zinc in their diets. Given that the participants were from these regions, it is possible that they may have had suboptimal zinc status, although this cannot be confirmed without direct measurements.

Additionally, these studies predominantly involved adolescent and young adult females, specifically middle-school and university students from regions where zinc deficiency is more prevalent. This focus raises concerns about regional and age-related biases, potentially limiting the generalizability of the findings. Unique cultural, dietary, and genetic factors in these areas, along with hormonal and physiological differences associated with youth, may influence the response to zinc supplementation and the severity of primary dysmenorrhea [59,60]. Therefore, the combination of these factors and the absence of detailed participant data raises questions about applying the results to older women or those from different geographical and cultural backgrounds.

## 5. Conclusions

In conclusion, zinc supplementation demonstrated a statistically significant pain-reducing effect in women with primary dysmenorrhea compared to placebo. The effect was significantly correlated with treatment duration, with sustained supplementation leading to greater pain reduction. Lower doses, such as 7 mg/day, of elemental zinc are sufficient to achieve significant pain relief without increasing the risk of adverse events. These findings support the consideration of zinc supplementation as a practical and safe approach to managing primary dysmenorrhea, potentially improving the quality of life for many women. Further research is necessary to validate these results in diverse populations and to investigate the long-term effects of zinc supplementation on primary dysmenorrhea.

## Figures and Tables

**Table 1:** Summary of the trials examining the effect of zinc supplementation on managing primary dysmenorrhea among the participants.

First Author and Year	Country	Population	Participants (Female)	Age ^1^	Study Design	Allocation Concealment	Randomization	Funding/Grants/Support
Kashefi 2014 [23]	Iran	Healthy subjects with PD	Ginger: 48 ^2^ Zinc sulfate: 56 Placebo: 46	17 ± 0.43 ^2^	RCT, double-blind	Not mentioned	Random table	Bojnurd University of Medical Science
Sangestani 2015 [24]	Iran	Healthy subjects with PD	Zinc sulfate: 34 ^2,3^ Placebo: 32	21.5 ± 2.5 ^2,3^ 21.7 ± 2.2	RCT, double-blind	Not mentioned	Not mentioned	N/A
Zekavat 2015 [25]	Iran	Healthy subjects with PD	Zinc sulfate: 60 ^2,3^ Placebo: 60	15.2 ± 1.7 ^2,3^ 14.8 ± 2.1	RCT, double-blind	Concealed envelope	Computer-based random digit generator	N/A
Teimoori 2016 [26]	Iran	Healthy subjects with PD	Zinc sulfate + mefenamic acid: 100 ^2,3^ Placebo + mefenamic acid: 100	21.6 ± 2.1 ^2,3^ 21.3 ± 2.0	RCT, double-blind	Not mentioned	Computer-based random digit generator	N/A
Safdar 2022 [13]	Pakistan	Healthy subjects with PD	Zinc gluconate: 50 ^2,3^ Placebo: 50	21.3 ± 1.86 ^2,3^ 20.78 ± 2.01	RCT, single-blind	Not mentioned	Lottery method	N/A
Obiagwu 2023 [12]	Nigeria	Healthy subjects with PD	Zinc sulfate: 50 ^3^ Placebo: 50	15.89 ± 1.54 ^3^	RCT, double-blind	Independent third party with concealed envelope	Random number table	N/A

PD, primary dysmenorrhea; RCT, randomized controlled trial. ^1^ Age is provided in the form of mean ± standard deviation. ^2^ Allocated participants. ^3^ Per-protocol participants.

**Table 2:** Summary of the zinc supplement interventions administered in the treatment arms of the included trials.

First Author and Year	Population	Duration	Zinc Supplement/Manufacturer	Daily Zinc Dose ^1^ (Per-Protocol N)	Control (Per-Protocol N)	Pain Measurement (Score Range)	AE Associated with Zinc Supplement Withdrawal
Kashefi 2014 [23]	Healthy high-school students with PD	1 and 2 months	Zinc sulfate capsules/Department of Industrial Pharmacy in the Faculty of Pharmacy (Iran)	150 mg/day ^2^ (53)	Matching placebo (42)	Pain Visual Analog Scale (0–10)	Not mentioned
Sangestani 2015 [24]	Healthy university students with PD	1 month	Zinc sulfate/Not mentioned	30 mg/day ^3^ (34)	Matching placebo (32)	Pain Visual Analog Scale (0–100)	No
Zekavat 2015 [25]	Adolescent females with PD	1, 2, and 3 months	Zinc sulfate capsules/Alhavi Drug Company (Iran)	15 mg/day ^4^ (60)	Matching placebo (60)	Pain Visual Analog Scale (0–10)	No
Teimoori 2016 [26]	Healthy university students with PD	3 months	Zinc sulfate/Not mentioned	50 mg/day ^5^ + mefenamic acid 750 mg/day (100)	Matching placebo + mefenamic acid 750 mg/day (100)	Pain Visual Analog Scale (0–10)	No
Safdar 2022 [13]	Healthy women with PD	3 and 6 months	Zinc gluconate/Not mentioned	7 mg/day ^6^ (50)	Matching placebo (50)	Pain Visual Analog Scale (0–10)	No
Obiagwu 2023 [12]	Healthy high-school students with PD	1, 2, and 3 months	Zinc sulfate/Chi Pharmaceutical Company (Nigeria)	9.2 mg/day ^7^ (50)	Matching placebo (50)	Pain Visual Analog Scale (0–10)	No. All withdrawals were not associated with the study intervention

AE, adverse event; PD, primary dysmenorrhea. ^1^ Zinc supplement dosages were standardized to elemental zinc, with zinc sulfate containing 23% and zinc gluconate 14.3% elemental zinc [27]. ^2^ Three times a day for four days, starting from the day before menstrual bleeding to the third day of menstrual bleeding. ^3^ Twice per day for a period of four days before the onset of menstruation. ^4^ One capsule per night during the intervention. ^5^ Zinc sulfate capsules once daily and mefenamic acid capsules (250 mg) three times daily, starting three days before menstruation and continuing for three days after. ^6^ Once daily for seven days, starting from five days before the estimated date of menstruation and two days after the onset of menstruation. ^7^ Once daily for three days, starting on the first day of menstruation and continuing until the third day of menstrual bleeding.

**Table 3:** Detailed quality assessment of included studies using the Cochrane Risk of Bias 2 tool.

First Author	Year	Randomization Process	Intervention Adherence	Missing Outcome Data	Outcome Measurement	Selective Reporting	Overall RoB
Kashefi [23]	2004	S ^1^	L	S ^2^	L	L	S
Sangestani [24]	2015	S ^1^	L	L	L	L	S
Zekavat [25]	2015	L	L	L	L	L	L
Teimoori [26]	2016	S ^1^	L	L	L	L	S
Safdar [13]	2022	S ^1^	L	L	L	L	S
Obiagwu [12]	2023	L	L	L	L	L	L

^1^ The studies did not provide allocation concealment details. ^2^ The study had a moderate proportion of missing data (7 out of 102 participants), with unclear reasons for dropout and insufficient handling of missing data. H, high risk of bias; S, some risk of bias; L, low risk of bias; RoB, risk of bias.

## Supplementary Materials

The following supporting information can be downloaded at: https://www.mdpi.com/article/10.3390/nu16234116/s1, Table S1: PRISMA checklist; Table S2: Keywords and search results in different databases; Table S3: Criteria for study selection in meta-analysis; Table S4: Excluded studies and reasons [33,61,62]; Table S5: Details of data extraction from included randomized controlled trials.
