# Supplementary material for: Efficacy of Zinc Supplementation in the Management of Primary Dysmenorrhea: A Systematic Review and Meta-Analysis
Source: Nutrients. 2024 Nov 28;16(23):4116. doi: 10.3390/nu16234116 (PMC11643716; doi:10.3390/nu16234116)
Supplement: Supplementary file 1 [file nutrients-16-04116-s001.zip › nutrients-3295345-supplementary.pdf]

Supplementary Tables

Table S1. PRISMA Checklist

Section and Topic	#	Checklist item	Location
**TITLE**			
Title	1	Identify the report as a systematic review.	Title
**ABSTRACT**			
Abstract	2	See the PRISMA 2020 for Abstracts checklist.	Abstract
**INTRODUCTION**			
Rationale	3	Describe the rationale for the review in the context of existing knowledge.	Introduction
Objectives	4	Provide an explicit statement of the objective(s) or question(s) the review addresses.	Introduction
**METHODS**			
Eligibility criteria	5	Specify the inclusion and exclusion criteria for the review and how studies were grouped for the syntheses.	Methods
Information sources	6	Specify all databases, registers, websites, organisations, reference lists and other sources searched or consulted to identify studies. Specify the date when each source was last searched or consulted.	Methods
Search strategy	7	Present the full search strategies for all databases, registers and websites, including any filters and limits used.	Methods, Table S2
Selection process	8	Specify the methods used to decide whether a study met the inclusion criteria of the review, including how many reviewers screened each record and each report retrieved, whether they worked independently, and if applicable, details of automation tools used in the process.	Methods
Data collection process	9	Specify the methods used to collect data from reports, including how many reviewers collected data from each report, whether they worked independently, any processes for obtaining or confirming data from study investigators, and if applicable, details of automation tools used in the process.	Methods, Table S4
Data items	10a	List and define all outcomes for which data were sought. Specify whether all results that were compatible with each outcome domain in each study were sought (e.g., for all measures, time points, analyses), and if not, the methods used to decide which results to collect.	Methods, Table S4
	10b	List and define all other variables for which data were sought (e.g., participant and intervention characteristics, funding sources). Describe any assumptions made about any missing or unclear information.	Methods<br>Table 1-2, Table S4
Study risk of bias assessment	11	Specify the methods used to assess risk of bias in the included studies, including details of the tool(s) used, how many reviewers assessed each study and whether they worked independently, and if applicable, details of automation tools used in the process.	Methods
Effect measures	12	Specify for each outcome the effect measure(s) (e.g., risk ratio, mean difference) used in the synthesis or presentation of results.	Methods
Synthesis methods	13a	Describe the processes used to decide which studies were eligible for each synthesis (e.g., tabulating the study intervention characteristics and comparing against the planned groups for each synthesis (item #5)).	Methods, Figure 1, Table 1-2, Table S3
	13b	Describe any methods required to prepare the data for presentation or synthesis, such as handling of missing summary statistics, or data conversions.	Methods, Table S4
	13c	Describe any methods used to tabulate or visually display results of individual studies and syntheses.	Methods
	13d	Describe any methods used to synthesize results and provide a rationale for the choice(s). If meta-analysis was performed, describe the model(s), method(s) to identify the presence and extent of statistical heterogeneity, and software package(s) used.	Methods
	13e	Describe any methods used to explore possible causes of heterogeneity among study results (e.g., subgroup analysis, meta-regression).	Methods

	13f	Describe any sensitivity analyses conducted to assess robustness of the synthesized results.	Methods
Reporting bias assessment	14	Describe any methods used to assess risk of bias due to missing results in a synthesis (arising from reporting biases).	Methods, Figure 2, Table 3
Certainty assessment	15	Describe any methods used to assess certainty (or confidence) in the body of evidence for an outcome.	Methods
**RESULTS**			
Study selection	16a	Describe the results of the search and selection process, from the number of records identified in the search to the number of studies included in the review, ideally using a flow diagram.	Results, Figure 1, Table S2-S3
	16b	Cite studies that might appear to meet the inclusion criteria, but which were excluded, and explain why they were excluded.	Results, Table S3
Study characteristics	17	Cite each included study and present its characteristics.	Results, Table 1-2
Risk of bias	18	Present assessments of risk of bias for each included study.	Figure 2, Table 3
Results of individual studies	19	For all outcomes, present, for each study: (a) summary statistics for each group (where appropriate) and (b) an effect estimates and its precision (e.g., confidence/credible interval), ideally using structured tables or plots.	Figure 3-8
Results of syntheses	20a	For each synthesis, briefly summarise the characteristics and risk of bias among contributing studies.	Results, Table 3
	20b	Present results of all statistical syntheses conducted. If meta-analysis was done, present for each the summary estimate and its precision (e.g., confidence/credible interval) and measures of statistical heterogeneity. If comparing groups, describe the direction of the effect.	Results, Figure 3-9
	20c	Present results of all investigations of possible causes of heterogeneity among study results.	Results, Figure 3-8
	20d	Present results of all sensitivity analyses conducted to assess the robustness of the synthesized results.	Results, Figure 7
Reporting biases	21	Present assessments of risk of bias due to missing results (arising from reporting biases) for each synthesis assessed.	Figure 2, Table 3
Certainty of evidence	22	Present assessments of certainty (or confidence) in the body of evidence for each outcome assessed.	Figure 3-9
**DISCUSSION**			
Discussion	23a	Provide a general interpretation of the results in the context of other evidence.	Discussion
	23b	Discuss any limitations of the evidence included in the review.	Discussion
	23c	Discuss any limitations of the review processes used.	Discussion
	23d	Discuss implications of the results for practice, policy, and future research.	Discussion
**OTHER INFORMATION**			
Registration and protocol	24a	Provide registration information for the review, including register name and registration number, or state that the review was not registered.	Methods
	24b	Indicate where the review protocol can be accessed, or state that a protocol was not prepared.	Methods, Table S2-S3
	24c	Describe and explain any amendments to information provided at registration or in the protocol.	Methods, Table S2-S3
Support	25	Describe sources of financial or non-financial support for the review, and the role of the funders or sponsors in the review.	Funding
Competing interests	26	Declare any competing interests of review authors.	Conflicts of Interest
Availability of data, code and other materials	27	Report which of the following are publicly available and where they can be found: template data collection forms; data extracted from included studies; data used for all analyses; analytic code; any other materials used in the review.	Results, Table S2-S4

Table S2. Keywords and search results in different databases

Database	Keyword	Filter	Date	Results
PubMed	(“Zinc Supplementation” OR “Zinc therapy” OR “Zinc sulfate” OR “Oral zinc”) AND (“Primary Dysmenorrhea” OR “Menstrual pain” OR “Menstrual cramps” OR “Period pain” OR “Dysmenorrhoea”) AND (“Randomized Controlled Trial” OR “Clinical trial” OR “RCT” OR “Randomised trial”)	Randomized Controlled Trial	May 15, 2024	3
Embase	(“Zinc Supplementation” OR “Zinc therapy” OR “Zinc sulfate” OR “Oral zinc”) AND (“Primary Dysmenorrhea” OR “Menstrual pain” OR “Menstrual cramps” OR “Period pain” OR “Dysmenorrhoea”) AND (“Randomized Controlled Trial” OR “Clinical trial” OR “RCT” OR “Randomised trial”)	NA	May 15, 2024	5
Cochrane Library	(“Zinc Supplementation” OR “Zinc therapy” OR “Zinc sulfate” OR “Oral zinc”) AND (“Primary Dysmenorrhea” OR “Menstrual pain” OR “Menstrual cramps” OR “Period pain” OR “Dysmenorrhoea”) AND (“Randomized Controlled Trial” OR “Clinical trial” OR “RCT” OR “Randomised trial”)	NA	May 15, 2024	7
Web of Science	(“Zinc Supplementation” OR “Zinc therapy” OR “Zinc sulfate” OR “Oral zinc”) AND (“Primary Dysmenorrhea” OR “Menstrual pain” OR “Menstrual cramps” OR “Period pain” OR “Dysmenorrhoea”) AND (“Randomized Controlled Trial” OR “Clinical trial” OR “RCT” OR “Randomised trial”)	NA	May 15, 2024	1
Google Scholar databases	(“Zinc Supplementation” OR “Zinc therapy” OR “Zinc sulfate” OR “Oral zinc”) AND (“Primary Dysmenorrhea” OR “Menstrual pain” OR “Menstrual cramps” OR “Period pain” OR “Dysmenorrhoea”) AND (“Randomized Controlled Trial” OR “Clinical trial” OR “RCT” OR “Randomised trial”)	NA	May 15, 2024	278

NA: not applied

Table S3. Criteria for study selection in meta-analysis

Criteria type	Description	Explanation
Inclusion criteria	Randomized controlled trials (RCTs)	This meta-analysis includes only randomized controlled trials (RCTs) to ensure high evidence quality, as RCTs minimize bias and provide reliable results for efficacy evaluation.
	Studies evaluating the efficacy of zinc supplementation in primary dysmenorrhea	The studies must focus on the efficacy of zinc supplementation in managing primary dysmenorrhea, addressing the specific condition under investigation.
	Studies reporting outcomes related to pain severity	Studies included should report outcomes that directly measure pain severity, as this is the primary outcome of interest in assessing the effectiveness of zinc supplementation.
Exclusion criteria	Studies involving secondary dysmenorrhea	Studies focused on secondary dysmenorrhea are excluded to maintain consistency and avoid confounding factors specific to secondary causes of dysmenorrhea.
	Observational studies, reviews, and case reports	Observational studies, reviews, and case reports are excluded to focus on high-quality evidence from RCTs, as these types of studies often have lower reliability for efficacy evaluation.
	Studies with incomplete data or non-comparable outcomes	Studies with incomplete or non-comparable data are excluded to ensure accurate and meaningful meta-analysis results, as incomplete data can lead to biased or unreliable conclusions.

Table S4. Excluded studies and reasons

Citations	Reasons
Farrah, A.M.; Halim, B.; Kaban, Y. Effectiveness of Zinc Supplementation in Treating Dysmenorrhea. Bali Medical Journal **2017**, 6, 34–37, doi:10.15562/bmj.v6i1.380. [61]	An experimental study, using one group pre and post-test design<br>Not a randomized trial
Sundari, L.P.R.; Adiputra, N.; Adiatmika, I.P.G.; Dinata, I.M.K. Oral Administration of Zinc Capsule for 4 Days before Menstrual Period Decreases Prostaglandin (PGF 2 α) Level and Pain Intensity in Women with Primary Dysmenorrhea. Int. J. Sci. Res. (Raipur) **2017**, 6, 1081–1084, doi:10.21275/ART20171702. [33]	An experimental study, using one group pre and post-test design<br>Not a randomized trial
Rao, R.; Nambiar, J.; Pai, M. A Prospective Interventional Study for the Effect of Zinc Sulphate on Pain Severity and Duration in Primary Dysmenorrhea. Indian Obstetrics and Gynaecology **2020**, doi:10.22541/au.159285493.39240825. [62]	A prospective interventional study<br>Not a randomized trial

Table S5. Details of data extraction from included randomized controlled trials

First Author & Year	Details of data extraction from included trials
Kashefi 2014	1. The age of allocated participants was from Results – Second paragraph.<br>2. Allocated participant numbers were from Figure 1.<br>3. The treatment-effect data was extracted from Table 2.<br>4. The data of side effects was extracted from Table 3.
Sangestani 2015	1. Participants' age and numbers were from Table 1.<br>2. The treatment-effect data was extracted from Table 2.<br>3. The visual analog scale, originally ranging from 0 to 100, was converted to a 0 to 10 scale using a simple scaling method by dividing the mean and standard deviation by 10.
Zekavat 2015	1. Participant' age was from Table 1.<br>2. Participant's numbers were from Figure 1.<br>3. The treatment-effect data was extracted from Table 2.
Teimoori 2016	1. Participants' age was extracted from Results – First paragraph.<br>2. Participants' numbers were from Table 2.<br>3. The treatment-effect data was extracted from Table 1.
Safdar 2022	1. Participants' age was from Results – First paragraph.<br>2. Participants' numbers and the data of side effects were from Figure 2 and Figure 2 was magnified and printed out then measured to improve measurement precision.<br>3. The treatment-effect data was from Table 1.
Obiagwu 2023	1. Participant' numbers were from Figure 1.<br>2. The age of the study population was extracted from Discussion – First paragraph.<br>3. The treatment-effect data was extracted from Table 4.<br>4. The data of side-effects was extracted from Table 5.
